# Intelligent tumor tissue classification for Hybrid Health Care Units

**DOI:** 10.3389/fmed.2024.1385524

**Published:** 2024-06-26

**Authors:** Muhammad Hassaan Farooq Butt, Jian Ping Li, Jiancheng (Charles) Ji, Waqar Riaz, Noreen Anwar, Faryal Farooq Butt, Muhammad Ahmad, Abdus Saboor, Amjad Ali, Mohammed Yousuf Uddin

**Affiliations:** ^1^School of Computer Science and Engineering, University of Electronic Science and Technology of China, Chengdu, China; ^2^Institute of Intelligent Manufacturing, Shenzhen Polytechnic University, Shenzhen, China; ^3^Shenzhen Institutes of Advanced Technology, Chinese Academy of Sciences, Shenzhen, China; ^4^Computer Engineering and Software Engineering Department, Polytechnique Montreal, Montreal, QC, Canada; ^5^Islamabad Medical Complex, NESCOM Hospital, Islamabad, Pakistan; ^6^Department of Computer Science, National University of Computer and Emerging Sciences, Chiniot-Faisalabad Campus, Chiniot, Pakistan; ^7^Department of Computer Science and Software Technology, University of Swat, Saidu Sharif, Pakistan; ^8^Department of Information Systems, College of Computer Engineering and Sciences, Prince Sattam Bin Abdulaziz University, Alkharj, Saudi Arabia

**Keywords:** hyperspectral imaging classification, Sharpened Cosine Similarity, deep learning, tumor tissues, Hybrid Health Care

## Abstract

**Introduction:**

In the evolving healthcare landscape, we aim to integrate hyperspectral imaging into Hybrid Health Care Units to advance the diagnosis of medical diseases through the effective fusion of cutting-edge technology. The scarcity of medical hyperspectral data limits the use of hyperspectral imaging in disease classification.

**Methods:**

Our study innovatively integrates hyperspectral imaging to characterize tumor tissues across diverse body locations, employing the Sharpened Cosine Similarity framework for tumor classification and subsequent healthcare recommendation. The efficiency of the proposed model is evaluated using Cohen's kappa, overall accuracy, and f1-score metrics.

**Results:**

The proposed model demonstrates remarkable efficiency, with kappa of 91.76%, an overall accuracy of 95.60%, and an f1-score of 96%. These metrics indicate superior performance of our proposed model over existing state-of-the-art methods, even in limited training data.

**Conclusion:**

This study marks a milestone in hybrid healthcare informatics, improving personalized care and advancing disease classification and recommendations.

## 1 Introduction

Our research explores the utilization of hyperspectral imaging (HI) to revolutionize tumor tissue classification in various body regions, aiming to impact the medical field significantly. This approach promises to refine diagnostic accuracy and pave the path for more personalized treatment plans. Taking a step toward the era of highly personalized, adequate healthcare, our study aims to enhance patient care. The reason HI is utilized for disease diagnosis is grounded in the understanding that changes in tissue's optical properties, stemming from morphological and biochemical alterations during disease progression, can be detected (1). For instance, rapid cell division in malignant cells leads to increased metabolic enzyme levels and the formation of new vessels through angiogenesis to meet the demand for nutrients and oxygen (2).

HI capitalizes on these changes to identify lesions and abnormal tissue without needing histological examination, saving time and improving treatment efficacy. Biopsy samples, which are stable and easily obtained from patients, implement scanning HI feasible. Recent studies have explored correlations between HI and histological examination results to validate HI as an accurate disease diagnostic tool. Various tissues, including the breast (3), liver (4), brain (5), kidney (6), stomach (7), head and neck (8), and thyroid gland (9), have been investigated, demonstrating HI's capability for disease diagnosis. The complexity of HI is addressed by employing artificial intelligence, which exhibits comparable diagnostic accuracy compared to histology.

One notable advantage of HI-based disease diagnosis is its ability to directly examine biopsy tissue during surgery. Unlike histology, which typically takes hours, HI can analyze tissue within minutes. This rapid analysis enables real-time assessment of resection margins to check for residual tumor tissues. In a study, HI successfully identified breast cancer from excised breast tissue during surgery with an accuracy exceeding 84% (10). Additionally, HI has found application in identifying blood cells, showcasing its potential to delineate abnormal tissue without relying on biochemical techniques (11). These applications underscore the capacity of HI to support swift and accurate decision-making in clinical settings. Our research contributes significantly to the field in addressing the pressing need for more adaptable and precise tumor classification in healthcare diagnostics. The following points outline the key contributions made in this study:

- Versatile tumor classification: introduces a hyperspectral imaging-based classifier offering location-independent and adaptable tumor classification, surpassing the limitations of existing methods.
- Sharpened Cosine Similarity (SCS): SCS is proposed as an innovative technique within the hyperspectral imaging classification framework, demonstrating superior precision and efficiency for tumor classification, especially under limited training data.
- Empirical evaluation: provides a rigorous empirical evaluation of the proposed model, substantiating its superior performance through metrics like Cohen's kappa, overall accuracy, and f1-score.
- Hybrid Health Care (HHC) integration: applies hyperspectral imaging classification within HHC Units, contributing to personalized and effective medical care solutions with broader implications for healthcare informatics.

## 2 Literature review

Traditional imaging techniques like Magnetic Resonance Imaging (MRI) (12), Computed Tomography (CT) Scans (13), Positron Emission Tomography (PET) Scans (14), Functional MRI (fMRI) (15), and Magnetic Resonance Spectroscopy (MRS) (16) have their own set of challenges in tumor detection (17). While these methods are indispensable, their specificity to specific tumor types hinders widespread application. Furthermore, implementing advanced deep learning algorithms presents scalability and real-time processing issues in clinical environments (18). Addressing these limitations, our approach offers a more versatile and computationally efficient alternative, enhancing its potential for clinical integration.

Elaborating on existing imaging modalities, MRI stands out for its high sensitivity (90%–95%) in brain tumor detection but grapples with the risk of false results and limitations in pinpointing specific tumor types or smaller lesions (19). CT Scans, utilizing X-rays, exhibit a sensitivity range of 60%–90% and a specificity of ~90%. Still, the method is constrained by radiation risks and less detailed soft tissue imaging (20). PET Scans employing ionizing radiation show varying sensitivity (70%–90%) and reasonable specificity (80%–90%), yet are subject to sensitivity limitations due to tumor characteristics and tracer use (21). fMRI, indicating brain activity through blood flow, offers high sensitivity (80%–90%) and specificity in identifying key brain areas but is susceptible to motion artifacts and variable interpretation (22). MRS provides a window into the biochemical makeup of tissues, yielding crucial data on tumor metabolism and types (23). Each modality contributes uniquely to tumor diagnosis, balancing specific advantages and inherent challenges.

Tumors, formed when cells behave abnormally, exhibit a range of sizes and can emerge anywhere in the body. Genes mutation, whether inherited, acquired gradually, or induced by substances like alcohol and tobacco, transform cells into cancerous ones (24). Growing tumors can invade neighboring tissues, displace normal cells, and produce enzymes breaking down surrounding tissues. Local invasion occurs when tumors grow larger, and metastasis happens when cancer cells spread to other body parts through blood or lymphatics (25). Classification involves categorizing tumors broadly by tissue, organ, or system, specifically by type, grading based on cellular and structural features using the World Health Organization (WHO) system, and staging using the Tumor Node Metastasis (TNM) system (26). Solid neoplasms, including carcinomas, sarcomas, and lymphomas, are classified based on type. The WHO Classification of Tumors provides detailed insights into tumor histotypes across various organ systems (27). According to the WHO system, tumor grading assigns a numerical grade (1–3) based on cellular differentiation. Staging relies on the TNM system, considering the presence of distant metastases (M), lymph node involvement (N), and the size or extension of the primary tumor (T) (28).

The most common cause of cancer death among children under the age of 15 and the second fastest-growing cause of cancer death among those over the age of 65 are brain tumors, which originate in brain cells and may be benign or malignant (29). Gene defects, exposure to certain chemicals, and radiation therapy to the head increase the risk of these tumors (30). Gliomas, the most common type, form from neural cells, including astrocytomas and ependymomas (31). Other types, such as brain stem gliomas, optic nerve gliomas, primitive neuroectodermal tumors (PNET), medulloblastomas, craniopharyngiomas, and pineal region tumors, pose distinct challenges in terms of location and characteristics (32). Understanding these variations is crucial for tailored treatment approaches and underscores the complexity of brain tumor classification and detection.

Moreover, Lung carcinoma, or lung cancer (33), results from genetic mutations in airway cells triggered by factors like smoking (34). It manifests as non-small-cell lung cancer (85%) and small-cell lung cancer (15%) (35). Breast cancer originates from mutated breast cells, often spreading invasively, with common types being lobular, ductal carcinoma *in situ* (DCIS), and invasive ductal carcinoma (IDC) (36, 37). Meningiomas, arising from brain membranes, may compress nearby tissues, and their slow growth lacks a defined cause (38). HI stands poised to revolutionize tumor classification and identification by capturing unique optical properties associated with different tumor types (39). HI offers a non-invasive and potentially rapid method for precise diagnosis, contributing to improved treatment strategies and patient outcomes in lung, breast, and meningiomas.

## 3 Materials and methods

### 3.1 Dataset

**Figure 1:**
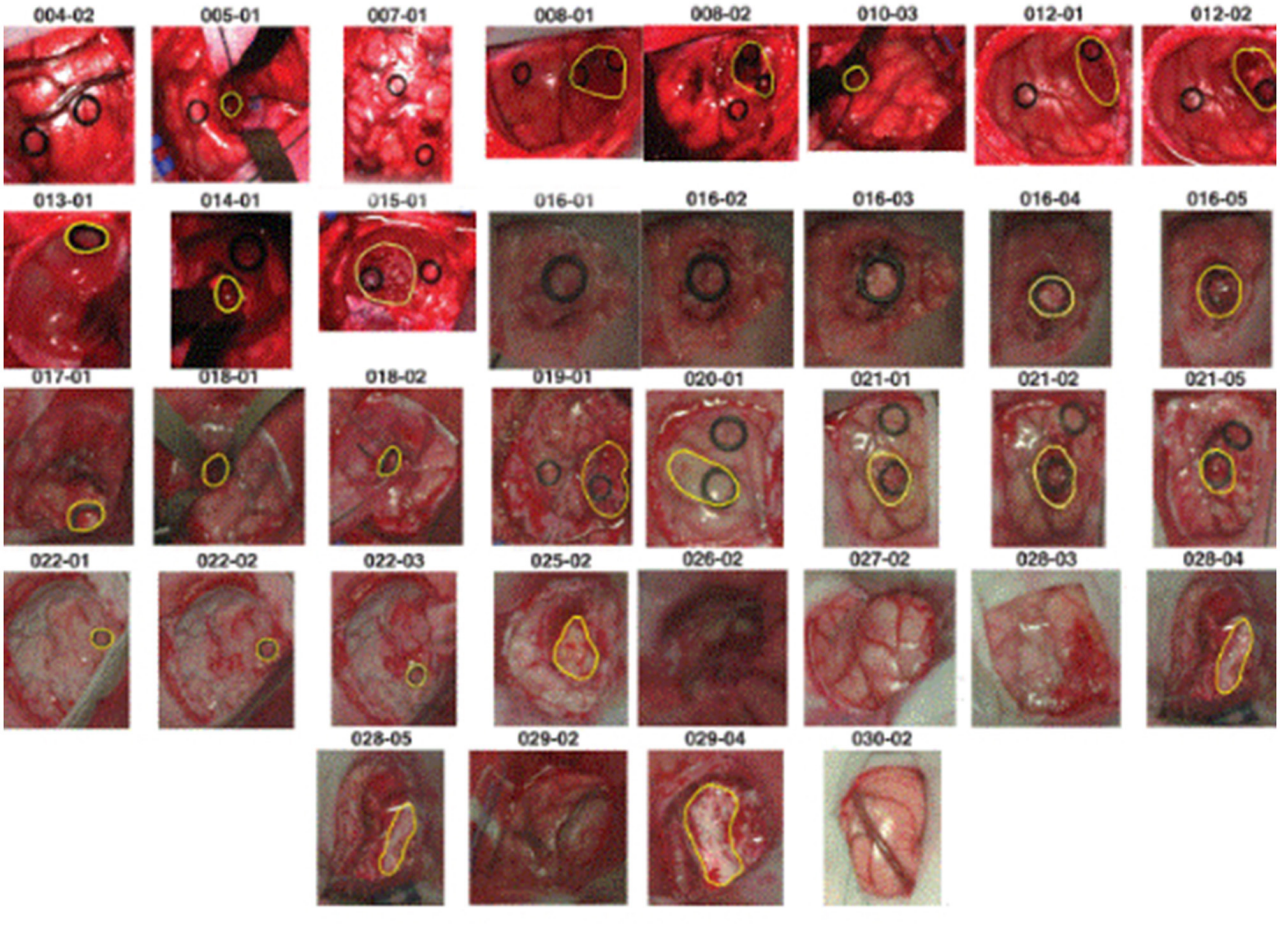
RGB representations of dataset images with PatientID-ImageID codes, delineating approximate tumor areas guided by neurosurgeon expertise and the IGS system.

The dataset we used to conduct experiments was initially collected and published by the *In-vivo* HS Human Brain database (40) comprising 36 *in-vivo* brain surface images from 22 unique patients. This labeled dataset includes tumor and normal tissue, blood vessels, and other irrelevant materials within the surgical scene (referred to as background). Tumor types are differentiated in the dataset, encompassing primary (grade IV glioblastoma and grade III and II anaplastic oligodendrogliomas) and secondary tumors (lung and breast). Additionally, RGB representations of hyperspectral cubes within the *in-vivo* hyperspectral human brain image database are presented in Figure 1. The dataset designates the approximate tumor area using a yellow line, aiding in identifying the rubber ring marker corresponding to pathological analyses of the tumor tissue. Patient ID and Image ID details in Table 1 offer a comprehensive overview, including image characteristics and the pathological diagnosis of each image. The total number of labeled pixels for each class and image is specified, addressing cases where certain images were diagnosed as specific tumor types without labeled tumor samples due to procedural challenges.

**Table 1:** Patient-specific image data and label distribution where “N” refers to “normal” tissue, “T” signifies “tumor tissue,” “BV” represents “blood vessels,” and “B” denotes the “background.”

**Patient ID**	**Image ID**	**Size**	**True labels**				**Diagnosis**
			**N**	**T**	**BV**	**B**	
004	02	389 × 345 × 826	✓	✗	✓	✓	Normal brain
005	01	483 × 488 × 826	✓	✗	✓	✓	Renal carcinoma (S)
007	01	582 × 400 × 826	✓	✗	✓	✗	Normal brain
008	01	460 × 549 × 826	✓	✓	✓	✓	Grade IV glioblastoma (P)
008	02	480 × 553 × 826	✓	✓	✓	✓	Grade IV glioblastoma (P)
010	03	460 × 549 × 826	✓	✗	✓	✓	Grade IV glioblastoma (P)
012	01	443 × 497 × 826	✓	✓	✓	✓	Grade IV glioblastoma (P)
012	02	445 × 498 × 826	✓	✓	✓	✓	Grade IV glioblastoma (P)
013	01	298 × 253 × 826	✓	✗	✓	✓	Lung carcinoma (S)
014	01	317 × 244 × 826	✗	✓	✓	✓	Grade IV glioblastoma (P)
015	01	376 × 494 × 826	✓	✓	✓	✓	Grade IV glioblastoma (P)
016	01	376 × 494 × 826	✓	✗	✓	✓	Normal brain
016	02	335 × 326 × 826	✓	✗	✗	✓	Normal brain
016	03	376 × 494 × 826	✓	✗	✓	✓	Normal brain
016	04	383 × 297 × 826	✓	✗	✓	✓	Grade IV glioblastoma (P)
016	05	414 × 292 × 826	✓	✗	✓	✓	Grade IV glioblastoma (P)
017	01	441 × 399 × 826	✓	✗	✓	✓	Grade IV glioblastoma (P)
018	01	479 × 462 × 826	✓	✗	✓	✓	Grade I glioblastoma (P)
018	02	510 × 434 × 826	✓	✗	✓	✓	Grade I glioblastoma (P)
019	01	601 × 535 × 826	✓	✗	✓	✓	Meningioma
020	01	378 × 330 × 826	✓	✓	✓	✓	Grade IV glioblastoma (P)
021	01	452 × 334 × 826	✓	✓	✓	✓	Breast carcinoma (S)
021	02	448 × 324 × 826	✓	✓	✓	✓	Breast carcinoma (S)
021	05	378 × 330 × 826	✓	✗	✓	✓	Breast carcinoma (S)
022	01	597 × 527 × 826	✓	✗	✓	✓	Grade III anaplastic oligodendroglioma (P)
022	02	611 × 527 × 826	✓	✗	✓	✓	Grade III anaplastic oligodendroglioma (P)
022	03	592 × 471 × 826	✗	✓	✗	✗	Grade III anaplastic oligodendroglioma (P)
025	02	473 × 403 × 826	✓	✓	✓	✓	Grade IV glioblastoma (P)
026	02	340 × 324 × 826	✓	✗	✓	✗	Normal brain
027	02	493 × 476 × 826	✓	✗	✓	✓	Normal brain
028	03	422 × 398 × 826	✓	✗	✓	✓	Normal brain
028	04	482 × 408 × 826	✗	✗	✗	✓	Lung adenocarcinoma (S)
028	05	482 × 390 × 826	✗	✓	✗	✗	Lung adenocarcinoma (S)
029	02	365 × 371 × 826	✓	✗	✓	✓	Normal brain
029	04	399 × 342 × 826	✗	✓	✗	✓	Grade II anaplastic oligodendroglioma (P)
030	02	382 × 285 × 826	✓	✗	✓	✓	Normal brain

The authors (40) mention the inherent challenges in acquiring *in-vivo* HI during neurosurgical procedures; the dataset primarily captures common tumor types over two years. The customized hyperspectral acquisition system, a preliminary demonstrator, is designed to capture tumor images on the surface or in easily focused deeper layers. The authors utilize a push broom camera for spatial scanning; the system's limitations include increased acquisition time and potential spatial coherence issues due to patient brain movement and procedural artifacts. As snapshot cameras offer real-time image acquisition but have fewer spectral bands than push-broom cameras, future investigations using high spectral resolution push-broom cameras are warranted. The dataset creation process by authors (40) addresses challenges from limited patient availability, presenting a preliminary database for exploring HI applications in tissue and tumor identification, tumor boundary delineation, and providing pertinent information for neurosurgeons. Their methodology leverages spectral characteristics guided by intraoperative MRI, surgeon expertise, and pathological analysis results. Subsequent data acquisition efforts are anticipated to broaden the database, encompassing more tumor types with detailed pathological descriptions.

### 3.2 HHC: AI tumor diagnostics

Our innovative methodology for tumor tissue classification within an HHC Unit unfolds with the patient's arrival at the facility. The initial phase involves a hyperspectral sensor scan, capturing intricate details of the patient's internal composition. This technology provides a comprehensive overview, laying the foundation for precise diagnosis. Following the hyperspectral scan, the acquired data undergoes processing through Factor Analysis. This step is crucial for dimension reduction, ensuring that the hyperspectral cube retains only relevant features essential for accurate classification. The processed data then traverses through the layers of our SCS model. As a breakthrough in tumor classification, the SCS model enhances precision, even when trained with limited data. This stage is pivotal for predicting and classifying tumor tissues, contributing to superior performance compared to existing models.

Once the classification is complete, the results are securely stored within the hospital's private records, ensuring data confidentiality. This stored information becomes a valuable resource for future reference and analysis. Integrated into the HHC Unit is a seamless access mechanism through Healthcare APIs. Healthcare professionals can leverage these APIs to access detailed reports and results related to tumor tissues. This integration streamlines the diagnostic process, providing a user-friendly interface for medical interpretation.

**Figure 2:**
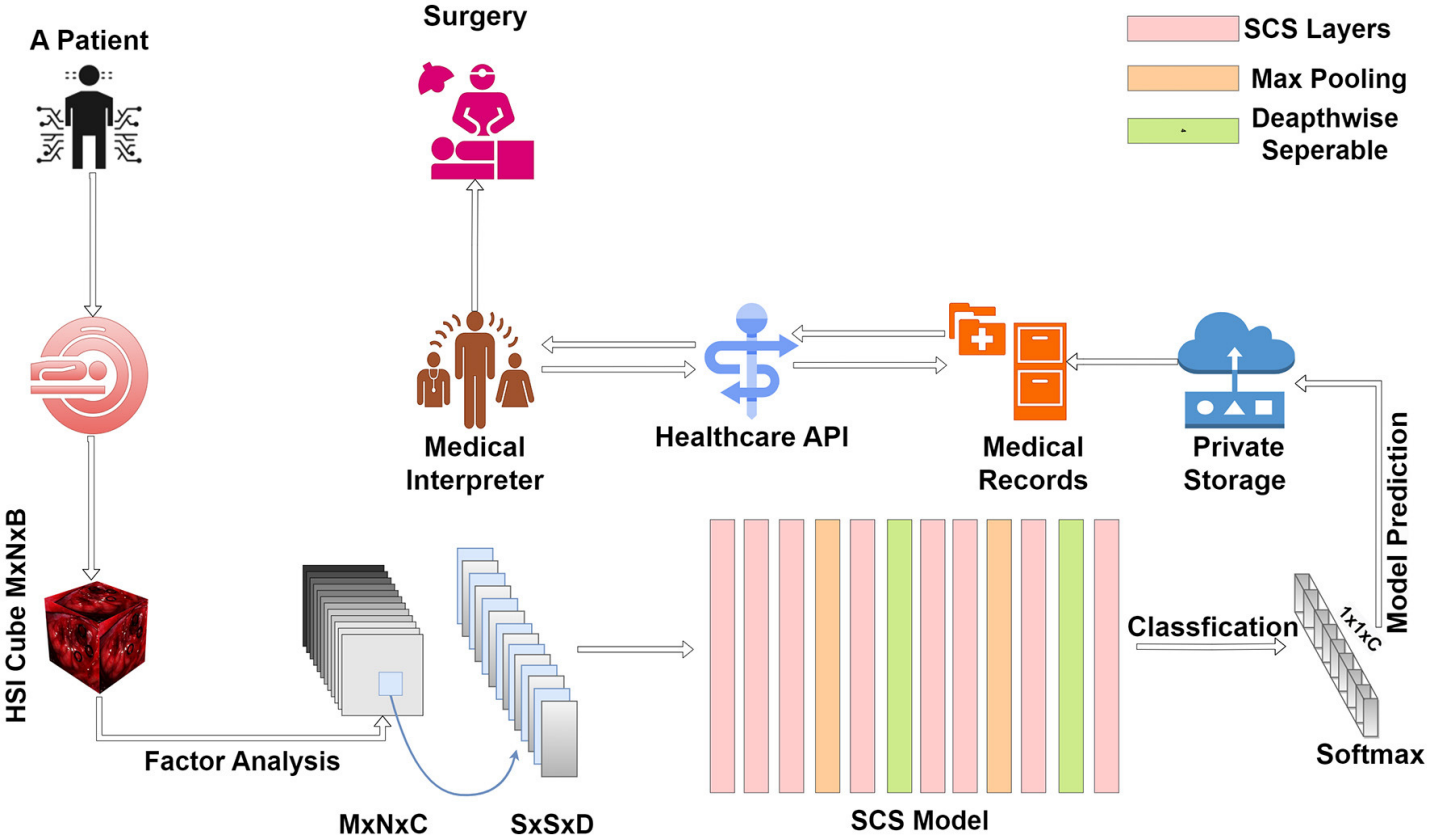
Streamlined tumor diagnosis in hybrid healthcare: a patient-centric approach from initial scan to tailored treatment.

The final phases of our methodology involve the medical interpreter within the Healthcare API, aiding healthcare professionals in interpreting results and making informed recommendations. These recommendations extend to surgical interventions and ongoing medical care, all tailored to the specific classification of tumor tissues and their respective locations. Figure 2 presents a comprehensive and patient-centric approach to tumor tissue classification within the HHC Unit. By seamlessly integrating hyperspectral imaging, Factor Analysis, and the innovative SCS model, we aim to revolutionize healthcare diagnostics and enhance the overall patient experience.

### 3.3 Proposed Sharpened Cosine Similarity method

HI represented as **X**∈ℝ^(*M*×*N*) × *B*^, where the dimensions (*M*×*N*) correspond to a specific area on the tissue surface and *B* denotes the total number of spectral bands in the HI. Each pixel within **X**, indicated as *x*_*ij*_ where *i* = 1, 2, …, *M* and *j* = 1, 2, …, *N*, is grouped into *C* unique tissue types, collectively expressed as **Y** = (*y*_1_, *y*_2_, …, *y*_*n*_). Moreover, every *x*_*ij*_∈**X** describes a tissue pixel through a spectral vector *x*_*ij*_ = [*x*_*i, j*, 1_, *x*_*i, j*, 2_, …, *x*_*i, j, B*_]∈**X**, containing a series of *B* spectral data points.

In the initial processing phase, spatial characteristics are emphasized by implementing a patch extraction method. This preliminary step involves the creation of a hyperspectral cube, ${\text{x}}_{i,j}\in {\mathbb{R}}^{\left(s\times s\right)\times D}$, encapsulating the area surrounding the focal pixel (*i, j*) over a region of dimensions *s*×*s*. This approach is instrumental in enhancing the model's ability to distinguish between different features by integrating spectral and spatial attributes. As such, the spectral-spatial cubes **x**_*i, j*_, drawn from the primary data and conforming to the dimensionality ℝ^(*s*×*s*) × *D*^, are consolidated into the dataset **X** in preparation for subsequent feature extraction processes. The concluding step involves the selection of training and testing samples across each distinct class.

In neural networks, the convolution operation involves a sliding dot product operation, symbolized as **w**·**x**_*ij*_, between an image patch **x**_*ij*_ and a filter **w**, which might miss crucial information due to its basic similarity measure. Enhancing this with normalization transforms the operation into cosine similarity, defined as $\frac{\text{w}\cdot {\text{x}}_{ij}}{\left||\text{w}||||{\text{x}}_{ij}|\right|}$. This is similar to calculating the cosine of the angle between vectors, utilizing Euclidean distance.

To address these limitations, Strided Cosine Similarity (SCS) was developed as expressed in Equation (1). It operates similarly to convolution but includes key differences. In standard convolution, the operation is a dot product **w**·**x**_*ij*_, while SCS involves normalizing the vectors. The normalization in SCS ensures the magnitude of vectors is unity before the dot product, leading to an expression like $\frac{\text{w}\cdot {\text{x}}_{ij}}{\left||\text{w}+\text{q}||||{\text{x}}_{ij}+\text{q}|\right|}$, where **q** is a small value to avoid numerical instability.

The similarity measure in SCS ranges between –1 and 1, indicating complete opposition or perfect alignment of the kernel and image patch, respectively. To mitigate the issue of small magnitudes, which can lead to noise inclusion, additional parameters are introduced in SCS, formulated as;


(1) $$\begin{array}{l} \text{SCS}\left(\text{w},{\text{x}}_{ij}\right)\text{=}\frac{\text{w}\cdot {\text{x}}_{ij}}{\left||\text{w}+\text{q}||||{\text{x}}_{ij}+\text{q}|\right|} \end{array}$$

Similar to conventional convolution in deep learning, SCS is a striding operation that extracts features from an image patch. However, it includes an additional step of magnitude normalization before the dot product, leading to what some literature refers to as Sharpened Cosine normalization. The effectiveness of SCS surpasses traditional convolutional processes in terms of speed due to fewer required parameters and the absence of normalization or activation functions.

In contrast to standard pooling, absolute max-pooling is employed in SCS for backpropagation filter updates, selecting the highest magnitude irrespective of the sign. The overall model with SCS is trained over 50 epochs, a batch size of 256, and a learning rate of 0.001. The learning rate significantly influences the model's learning rate, while momentum aids accuracy and speed. An root mean square prop and momentum-based optimizer, specifically the Adam optimizer, is utilized for its efficiency and computational advantages.

## 4 Experiment analysis

This section presents an overview of the evaluation metrics, baselines SOTA and implementation details.

### 4.1 Evaluation metrics

The results presented in this study are evaluated using the following metrics:

Kappa statistic: This statistical measure assesses the level of agreement between predicted classifications and ground-truth maps, as defined by Equation (2). In this equation, *A*_*o*_ represents the observed agreement, calculated using Equation (3), while *A*_*e*_ denotes the expected agreement, computed using Equation (4).


(2) $$\begin{array}{l} \kappa =\frac{A_{o}-A_{e}}{1-A_{e}} \end{array}$$

where,


(3) $$\begin{array}{l} A_{o}=\frac{TP+TN}{TP+FN+FP+TN} \end{array}$$

and,


(4) $$\begin{array}{l} \begin{array}{c} A_{e}=\left(\frac{FN+TN}{TP+FN+FP+TN}\times \frac{FP+TN}{TP+FN+FP+TN}\right)\\ \;+\frac{TP+FN}{TP+FN+FP+TN} \end{array} \end{array}$$

Here, *TP* and *FP* denote true positives and false positives, respectively, while *TN* and *FN* represent true negatives and false negatives.

Average accuracy (AA): AA signifies the average classification performance across different classes, as depicted in Equation (5).


(5) $$\begin{array}{l} AA=\frac{TP+TN}{TP+TN+FN} \end{array}$$

Overall accuracy (OA): OA is computed as the ratio of correctly classified examples to the total number of test examples, as defined by Equation (6).


(6) $$\begin{array}{l} OA=\frac{1}{N}\overset{N}{\underset{i=1}{\sum }}TP_{i} \end{array}$$

In the equations above, *TP* represents true positives, *FP* represents false positives, *TN* represents true negatives, and *FN* represents false negatives.

### 4.2 Baseline models

#### 4.2.1 Recurrent Neural Networks

The Recurrent Neural Networks (RNN) architecture (41) presents a blend of convolutional and fully connected layers within a Sequential model. Beginning with a Conv2D layer employing a 3 × 3 kernel and ReLU activation, the subsequent MaxPooling2D layer downsamples the spatial dimensions. Flattening the output precede a fully connected layer of 100 neurons, integrated with Batch Normalization and ReLU activation for regularization. With softmax activation, the final layer tailors the output to fit the specified number of classes. This design reflects a hybrid approach, incorporating convolutional operations followed by dense layers, offering flexibility for various applications in classification tasks.

#### 4.2.2 2-Dimensional Convolution Neural Network

The 2-Dimensional Convolution Neural Network (2D CNN) architecture (42) is structured within a Sequential model, featuring a Conv2D layer with a 3 × 3 kernel and ReLU activation, applied to input data of shape (window size, window size, kernel size). Subsequently, a MaxPooling2D layer down-samples spatial dimensions with a pooling size adjustment option. The flattened output leads to a fully connected layer with 100 neurons, supplemented by Batch Normalization and ReLU activation for regularization. The final layer, employing softmax activation, tailors the output to match the specified number of classes. This design reflects a standard 2D convolutional neural network suitable for diverse classification tasks with image data. Adjustments to the pooling size provide adaptability based on specific requirements.

#### 4.2.3 LeNet

The LeNet architecture, a seminal convolutional neural network devised by Yann LeCun in the 1990s, marked a breakthrough in computer vision (43). Comprising two convolutional layers with 5 × 5 filters and ReLU activation, each succeeded by average pooling; the network captures hierarchical features in the input. The subsequent dense layers, with 120 and 84 neurons, distill high-level representations. The final layer, employing softmax activation, tailors the output to the number of classes. LeNet's simplicity and efficacy laid the foundation for modern CNNs, influencing subsequent developments in image classification.

#### 4.2.4 Xception

The Xception architecture (44) is a 2D variant of the Xception neural network, known for its depth-wise separable convolutions and exceptional performance in image classification tasks. The model begins with an entry flow featuring a series of convolutional layers with batch normalization and ReLU activation. The residual block 1 introduces separable convolutions, preserving spatial information efficiently. The middle flow comprises eight repeated blocks, each containing three separable convolutional layers, facilitating feature extraction. The exit flow further refines features with a combination of separable convolutions and residual connections. The model concludes with a global average pooling layer and a dense layer with softmax activation, tailoring the output to the specified number of classes. The Xception architecture is designed to capture complex hierarchical features in image data, making it suitable for various image classification tasks. Adjustments to the number of filters and other parameters can be made based on specific requirements.

### 4.3 Implementation details

**Figure 3:**
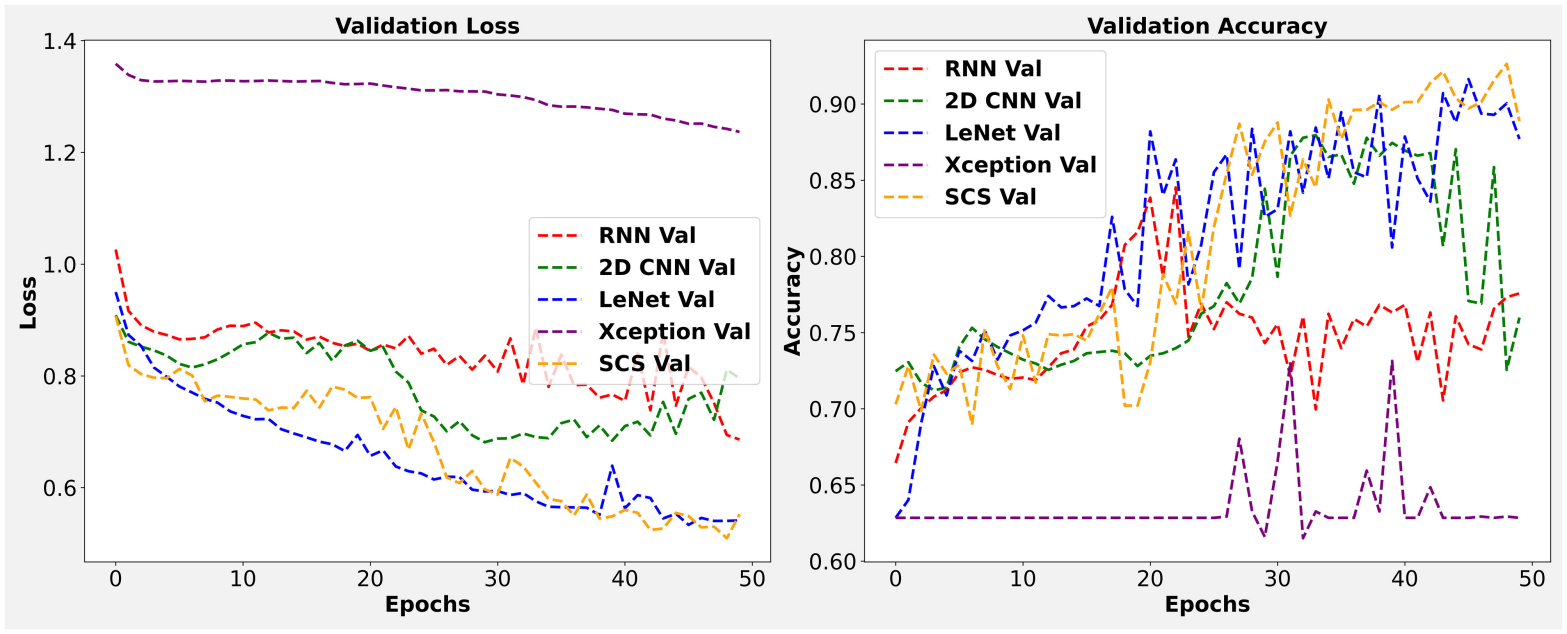
Visualization of validation loss and accuracy for 2D CNN, RNN, LeeNet, Xception and SCS.

For our empirical assessment, we utilized *in-vivo* HS Human Brain database which is already discussed in Section 3.1 and accessible on request on this https://hsibraindatabase.iuma.ulpgc.es/. This experiment used a Jupyter notebook running on an Intel 11th Gen processor and 32 GB of RAM. For all experiments, the training, validation, and test samples distribution was set at 15%, 15%, and 70%, respectively. To ensure an equitable comparison, all models, including the RNN, 2D CNN, LeeNet, Xception, and proposed SCS models, were executed simultaneously with a single, randomly chosen set of samples. The reported results were achieved using a patch size of 3 × 3, and the three most informative bands were identified through Factor Analysis (FA). Regarding training parameters, the models began with randomly initialized weights, which were subsequently optimized via backpropagation using the Adam optimizer and a softmax loss function. Figure 3 present a detailed analysis of the validation loss and accuracy for all models under consideration. In this study, we adhere to this principle by keeping these parameters uniform across all compared methods, including our SCS pipeline, within a single execution run.

## 5 Discussion

In this section, we conduct a twofold comparative analysis to evaluate the performance of our SCS pipeline for the Hybrid Healthcare Unit. Firstly, at the patient level, we assess the system's efficacy in providing personalized tumor tissue classifications and treatment recommendations. Subsequently, at the same tumor class level, we analyze the system's precision in distinguishing minute variations within specific classes. These comparative experiments aim to comprehensively understand the Hybrid Healthcare Unit's capabilities, addressing individual patient needs and the challenges within distinct tumor classes.

### 5.1 Comparative experiment—Class level

**Figure 4:**
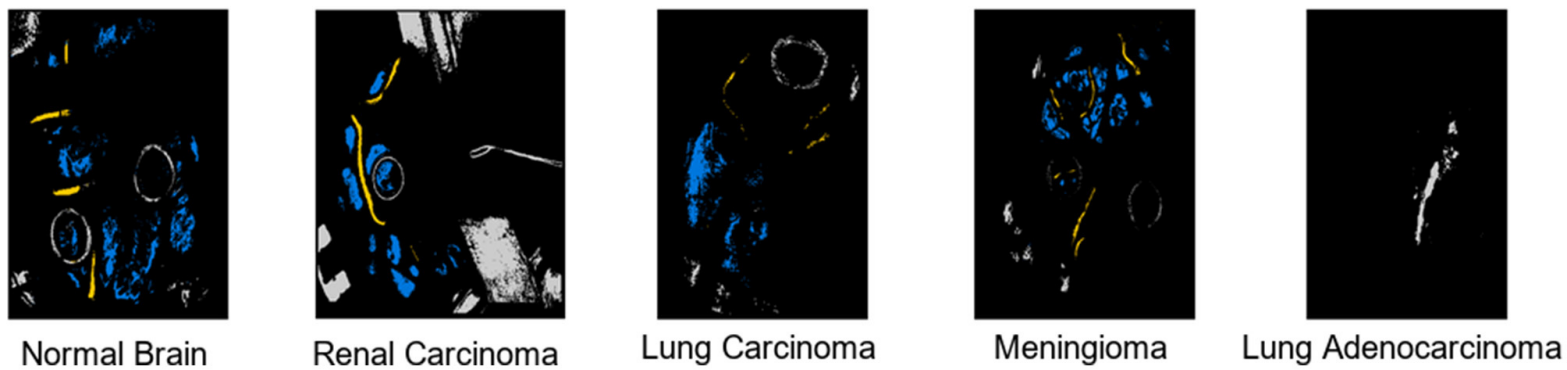
Output visualization of tumor tissues classification across different body locations.

We present a comprehensive performance analysis based on the F1-Score, comparing SOTA, including 2D CNN, RNN, LeeNet, Xception, and our proposed SCS across different patients. The objective of this comparative experiment class level is to evaluate and compare the performance of these models in accurately classifying different tissue types in HI as results discussed in Table 2. Across different patients, our SCS consistently achieves high accuracy in predicting tissue classes, as presented in the Table 3. Notably, for Patient IDs 005, 008, 022, 028, and 029, SCS achieves exceptional accuracy close to or at 100% in classifying normal tissue, hypervascularized tissue, and background classes. This demonstrates the model's robustness in handling diverse cases. In cases where tumor tissue is present, the SCS model also successfully achieves accurate predictions comparison with other models (2D CNN, RNN, LeeNet, and Xception 2D). The model's effectiveness in leveraging SCS-enhanced features for accurate tissue classification irrespective of body location are shown in classification map outputs (Figure 4). These sub-figures correspond to different tumor tissue types: Normal Brain, Renal Carcinoma, Lung Carcinoma, Meningioma, and Lung Adenocarcinoma. The model's ability to handle various tissue classes and consistent accuracy across different patients and images highlight its potential as a valuable tool in medical diagnostics, particularly for tumor tissue classification.

**Table 2:** Performance analysis of the SOTA models on each predicted class for different patient IDs and image IDs.

**Patient ID**	**Image ID**	**Classes**	**2D CNN**	**RNN**	**LeeNet**	**Xception**	**SCS model**
004	02	Normal tissue	0.88	0.86	**0.97**	0.77	0.96
				Tumor tissue	–	–	–	–	–
				Hypervascularized tissue	0.44	0.18	0.26	0.00	**0.82**
				Background	0.54	0.68	0.75	0.00	**0.77**
005	01	Normal tissue	0.85	0.87	0.95	0.72	**0.98**
				Tumor tissue	–	–	–	–	–
				Hypervascularized tissue	0.50	0.22	0.28	0.10	**0.98**
				Background	0.50	0.63	0.80	0.15	**0.86**
008	01	Normal tissue	0.82	0.85	0.89	0.82	**0.99**
				Tumor tissue	0.95	0.92	0.98	0.15	**1.00**
				Hypervascularized tissue	0.84	0.81	0.89	0.16	**0.99**
				Background	0.89	0.92	0.96	0.19	**1.00**
013	01	Normal tissue	**0.99**	0.89	**0.99**	0.95	**0.99**
				Tumor tissue	–	–	–	–	–
				Hypervascularized tissue	0.92	0.88	0.95	0.11	**1.00**
				Background	0.94	0.93	**0.99**	0.15	**0.99**
018	01	Normal tissue	**1.00**	**1.00**	**1.00**	0.98	**1.00**
				Tumor tissue	–	–	–	–	–
				Hypervascularized tissue	0.98	0.98	0.99	0.18	**1.00**
				Background	**1.00**	**1.00**	0.25	0.15	**1.00**
019	01	Normal tissue	0.97	0.87	**1.00**	0.91	**1.00**
				Tumor tissue	–	–	–	–	–
				Hypervascularized tissue	0.94	0.31	**0.99**	0.25	**0.99**
				Background	0.91	0.69	0.99	0.18	**1.00**
021	01	Normal tissue	0.93	0.26	0.98	0.95	**1.00**
				Tumor tissue	0.71	0.47	0.97	0.35	**0.99**
				Hypervascularized tissue	0.95	0.42	**1.00**	0.13	**1.00**
				Background	0.98	0.73	0.95	0.23	**0.98**
022	01	Normal tissue	1.00	0.80	**1.00**	0.89	**1.00**
				Tumor tissue	–	–	–	–	–
				Hypervascularized tissue	0.95	0.69	**1.00**	0.19	**1.00**
				Background	0.97	0.81	**1.00**	0.23	**1.00**
028	05	Normal tissue	–	–	–	–	–
				Tumor tissue	**1.00**	**1.00**	**1.00**	0.95	**1.00**
				Hypervascularized tissue	–	–	–	–	–
				Background	–	–	–	–	–
029	04	Normal tissue	–	–	–	–	–
				Tumor tissue	0.98	0.76	0.98	0.86	**1.00**
				Hypervascularized tissue	–	–	–	–	–
				Background	0.99	0.75	0.86	0.12	**1.00**

The bold values represent the Class-Wise Performance Analysis Based on F1-Score for Different Patient IDs and Image IDs.

**Table 3:** Comparative performance analysis of SOTA at patient and image level for each predicted class.

**Patient ID**	**Image ID**	**Classes**	**2D CNN**	**RNN**	**LeeNet**	**Xception**	**SCS model**
004	02	Kappa accuracy	49.93	47.66	71.67	30.02	**80.53**
				Overall accuracy	77.00	77.09	85.61	62.86	**89.91**
				Average accuracy	59.23	54.37	67.09	33.33	**86.25**
				F1-score	77	77	86	63	**90**
				Training time (seconds)	**10.15**	10.35	10.23	226.65	68.92
				Testing time (seconds)	0.75	0.78	**0.70**	4.68	2.04
005	01	Kappa accuracy	58.46	60.51	68.85	29.45	**91.98**
				Overall accuracy	66.77	63.92	70.35	35.25	**97.6**
				Average accuracy	61.66	57.33	67.66	32.33	**94.0**
				F1-score	68	59	70	34	**97**
				Training time (seconds)	**39.63**	41.60	42.93	254.75	83.45
				Testing time (seconds)	**1.71**	1.95	2.10	5.65	3.12
008	01	Kappa accuracy	84.60	84.0	89.84	31.47	98.45
				Overall accuracy	90.10	89.91	95.89	37.35	100.0
				Average accuracy	87.50	87.25	93.90	33.45	99.50
				F1-score	91	90	95	35	100
				Training time (seconds)	8.94	8.42	**7.30**	154.89	75.2
				Testing time (seconds)	**1.38**	2.91	1.63	2.84	1.73
013	01	Kappa accuracy	92.59	86.6	96.47	39.25	**99.45**
				Overall accuracy	93.97	92.9	98.6	41.79	**99.76**
				Average accuracy	95.16	90.4	97.66	40.33	**99.89**
				F1-score	95	90	98	42	**100**
				Training time (seconds)	**5.73**	7.94	6.76	107.21	80.72
				Testing time (seconds)	**0.23**	1.71	1.60	2.40	1.65
018	01	Kappa accuracy	99.23	99.51	72.12	41.62	**99.71**
				Overall accuracy	99.61	99.75	76.59	44.82	**99.85**
				Average accuracy	99.11	99.40	74.66	43.66	**99.38**
				F1-score	**100**	**100**	76	45	**100**
				Training time (seconds)	**36.57**	107.21	86.04	352.47	239.37
				Testing time (seconds)	**1.36**	1.60	1.40	4.37	1.83
019	01	Kappa accuracy	93.38	25.45	98.58	41.68	98.95
				Overall accuracy	95.88	61.83	99.11	45.23	99.50
				Average accuracy	86.93	43.01	98.45	44.66	99.33
				F1-score	96	62	99	46	100
				Training time (seconds)	**17.17**	95.1	38.46	537.42	354.81
				Testing Time (seconds)	**0.59**	2.89	1.43	6.85	4.29
021	01	Kappa accuracy	85.12	45.74	97.83	38.61	98.55
				Overall accuracy	90.99	48.39	98.91	42.35	99.75
				Average accuracy	89.25	45.19	97.56	41.50	99.25
				F1-score	90	47	98	41	100
				Training time (seconds)	**3.47**	5.41	38.46	37.42	34.18
				Testing time (seconds)	**1.54**	2.89	1.43	3.85	3.19
022	01	Kappa accuracy	97.86	65.68	**99.82**	43.12	99.80
				Overall accuracy	98.92	79.33	99.89	47.98	**99.90**
				Average accuracy	97.33	74.64	**99.90**	46.66	99.85
				F1-score	98	79	**100**	48	**100**
				Training time (seconds)	**11.80**	67.98	23.49	335.96	139.54
				Testing time (seconds)	**0.48**	1.54	0.85	3.38	1.24
028	05	Kappa accuracy	–	–	–	–	–
				Overall accuracy	**100**	**100**	**100**	97.42	**100**
				Average accuracy	**100**	**100**	**100**	95.36	**100**
				F1-score	**100**	**100**	**100**	95	**100**
				Training time (seconds)	**4.71**	6.45	9.08	96.41	43.18
				Testing time (seconds)	**0.20**	0.37	0.53	1.76	0.93
029	04	Kappa accuracy	–	–	–	–	–
				Overall accuracy	98.9	75.8	92.69	50.81	**100**
				Average accuracy	98.3	75.64	91.45	49.05	**100**
				F1-score	99	78	92	50	**100**
				Training time (seconds)	**3.91**	5.59	5.05	35.55	27.91
				Testing time (seconds)	**0.40**	0.68	0.43	1.86	1.12

The bold values represent the Comparative Performance Analysis of SOTA and SCS Models Across Various Evaluation Metrics.

### 5.2 Comparative experiment—Patient level

**Figure 5:**
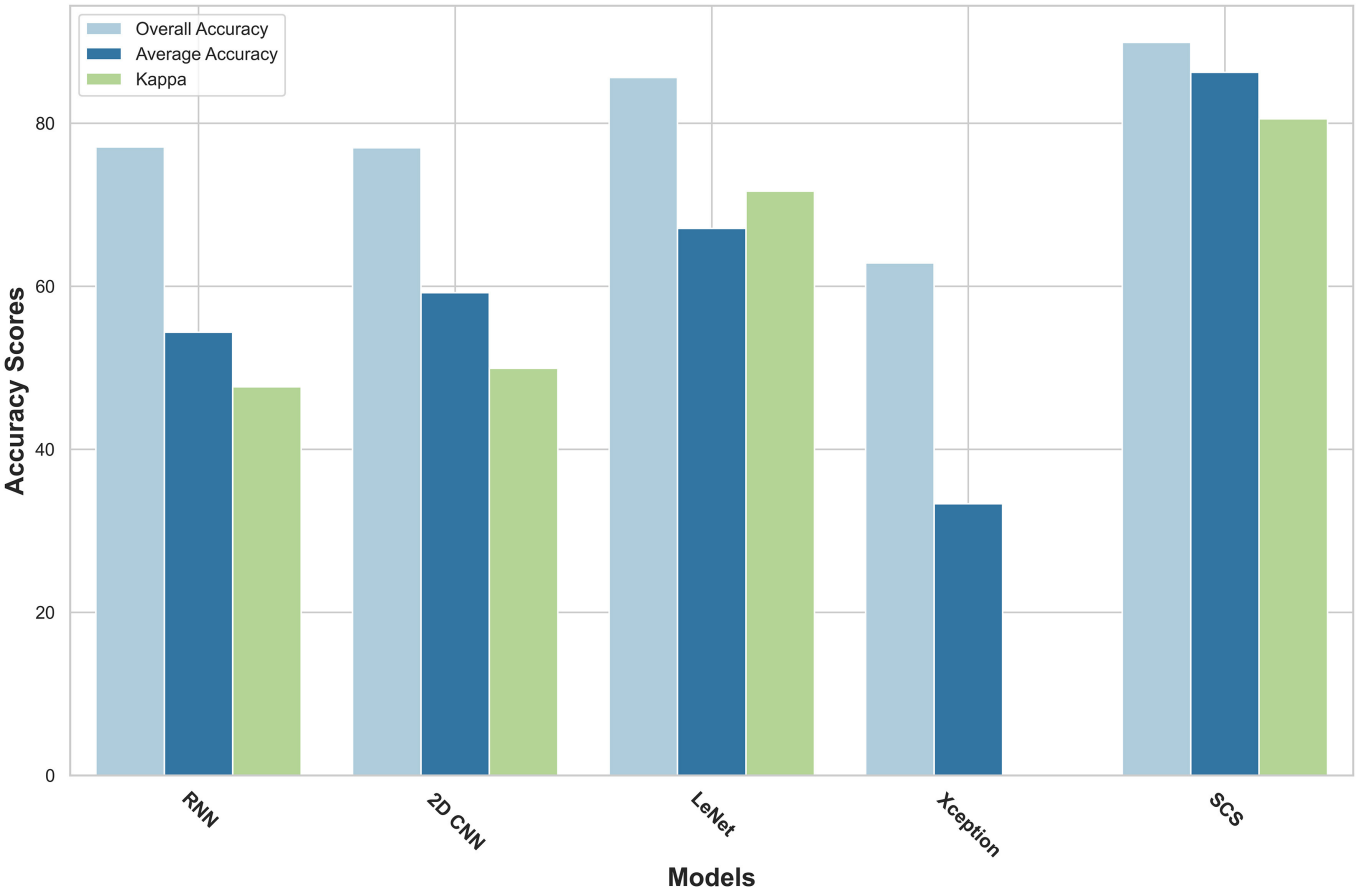
Visualization of evaluation metrics across SCS, RNN, 2D CNN, LeeNet, and Xception.

A detailed analysis of the performance of SOTA models on key metrics, including Kappa Accuracy, Overall Accuracy, Average Accuracy, F1-Score, Training Time, Testing Time, and Memory Consumption. Table 3 summarizes the performance metrics for each patient and their corresponding image IDs across various tissue classes. From the patient-level experiment, the SCS model consistently outperformed both models across multiple performance metrics. For instance, in Patient ID 004, the SCS model achieved a Kappa Accuracy of 80.53, surpassing 2D CNN (49.93), RNN (47.66), LeeNet (71.67) and Xception (30.02). Similar trends were observed regarding Overall Accuracy, Average Accuracy, and F1 score, where the SCS model consistently demonstrated superior performance across all patient IDs. Notably, in Patient ID 021, the SCS model achieved a Kappa Accuracy of 98.55, significantly surpassing 2D CNN (85.12), RNN (45.74), LeeNet (97.83), and Xception (38.61). SCS model's ability to consistently attain high accuracy, coupled with efficient training times and memory consumption, underscores its potential for accurate tissue classification in HI data, highlighting its value in practical medical applications. Although other models such as 2D CNN, RNN, and LeeNet have less training time, their accuracy is low compared to the SCS model; as we know, in deep learning, there is a trade-off between speed and accuracy. Figure 5 shows results underscore the superior performance of the SCS model across various metrics, indicating its efficacy in accurately classifying tissue types in HI data. The consistent out performance of the SCS model reaffirms its potential to enhance medical diagnostics and contribute to real-world applications.

## 6 Conclusion

Our research highlights the pivotal role of HI integrated with AI in advancing tumor tissue classification with the new Hybrid Health Care Units landscape. The innovative application of the Sharpened Cosine Similarity framework has proven highly effective, achieving remarkable performance metrics of 91.76% Cohen's kappa, 95.60% overall accuracy, and 94.29% f1-score. These results, surpassing current SOTA research even under limited training data, affirm our proposed model's robustness and potential clinical impact. The scarcity of specific hyperspectral medical data has been acknowledged as a challenge, emphasizing the need for ongoing efforts to expand and diversify datasets for further validation and generalization of our approach. However, the demonstrated superiority of our model in tumor classification positions it as a valuable tool for enhancing diagnostic capabilities in medical imaging. Future research could extend the proposed model by diversifying and expanding hyperspectral medical datasets for broader validation. Exploring real-time implementation in clinical settings and investigating additional AI techniques could enhance predictive capabilities. Furthermore, exploring broader applications beyond tumor classification, such as skin conditions, could maximize the model's utility. These efforts would advance healthcare informatics, improving diagnostic accuracy within Hybrid Health Care Units.

## Data availability statement

The datasets presented in this study can be found in online repositories. The names of the repository/repositories and accession number(s) can be found at: https://hsibraindatabase.iuma.ulpgc.es.

## Author contributions

MB: Visualization, Methodology, Conceptualization, Writing—review & editing, Writing—original draft. JL: Writing—review & editing, Supervision. JJ: Writing—review & editing, Supervision. WR: Writing—original draft, Funding acquisition. NA: Writing—original draft, Methodology, Formal analysis. FB: Writing—original draft, Validation, Data curation. MA: Writing—review & editing, Methodology, Conceptualization. AS: Writing—original draft, Visualization. AA: Writing—review & editing, Formal analysis, Validation. MU: Writing—review & editing, Supervision, Validation.
